# Design for 4D Printing of Biodegradable Shape Memory Polymers for Disposable UAV Systems

**DOI:** 10.3390/polym15173562

**Published:** 2023-08-27

**Authors:** Mary Ebeid, Sagil James

**Affiliations:** Department of Mechanical Engineering, California State University Fullerton, Fullerton, CA 92831, USA

**Keywords:** unmanned aerial vehicle, biodegradable shape memory polymers, 4D printing

## Abstract

The use of bio-based smart materials is vital for achieving the desired morphing characteristics while improving the efficiency of disposable Unmanned Aerial Vehicle (UAV) systems. Smart materials combine the structure and actuator into a single element without discrete moving parts, thereby minimizing the weight and mechanical complexity of the UAV system. Biodegradable smart materials facilitate the realization of smart actuation concepts that help in potentially improving system reliability and reducing the risk of potential component failure. However, the manufacturing of biodegradable smart materials is a huge challenge. Recent advances in 3D printing technologies have opened new possibilities for manufacturing biodegradable smart materials. The 3D printing technologies have been further extended to 4D printing, which essentially involves fabricating 3D smart structures. The goal of this research is to investigate the manufacturing challenges involved in the 4D printing of biodegradable smart polymer materials for disposable UAV systems. The manufacturing process consists of a combination of biodegradable smart polymer materials in conjunction with SLA-based 3D printing technology. The study will involve extensive theoretical investigations followed by experimental studies. The results of this study are expected to open up new possibilities for using biodegradable smart polymer materials at commercial scales.

## 1. Introduction

Unmanned Aerial Vehicles (UAVs) are aircraft that operate without human pilots on board. They are controlled either by autopilot systems or remotely from the ground [1]. UAVs are equipped with cameras and sensors to monitor their surroundings and flight paths. They have applications in various fields where human involvement is unsafe, such as traffic monitoring, exploration, firefighting, and maritime operations. The Coyote Unmanned Aircraft System (UAS) by Raytheon Technologies is a notable UAV used by the US Army for surveillance missions and by NOAA for hurricane tracking [2]. UAVs are designed to meet specific requirements based on their mission, including size, speed, and altitude capabilities [3]. This specialization creates limitations, as UAVs designed for certain speeds or altitudes may not perform well in different scenarios. Material choice is crucial for UAV design, as it needs to be strong yet lightweight. To address this, UAV wings are often designed as frameworks rather than rigid parts [4].

The complexity of wing design increases with the addition of components such as ribs, spars, and wing skin. Additional features such as high-lift devices (HLDs) further complicate the design, requiring hinges and actuators. These additions increase weight and manufacturing complexity [5]. To simplify manufacturing and reduce the number of parts, smart materials such as shape memory polymers (SMPs) can be used. SMPs can change shape in response to external stimuli, offering the potential to replace mechanical actuators and other components [6]. However, SMPs still have limitations that need to be addressed, such as the number of shape-change cycles they can successfully complete and their ability to fully recover their shape over time [7]. Despite these shortcomings, SMPs show promise in various industries, including aerospace, automotive, and biomedical sectors.

The development of bio-based polymers has brought about exciting opportunities for sustainable and eco-friendly technologies [8]. These biodegradable polymers are cost-effective and naturally degrade in the environment after a limited period of use [9,10]. The concept of biopolymers can be extended to biodegradable smart materials, combining the advantages of bio-based and smart materials. Biodegradable shape memory polymers have already found applications in fields such as medicine, robotics, and food [11,12]. While the manufacturing process for SMPs still requires extensive production and assembly, additive manufacturing, also known as 3D printing, has gained popularity for its ability to produce complex parts efficiently. However, the limitation of 3D printing is the difficulty of creating movable parts, which can be addressed through 4D printing [13]. By using smart materials such as shape memory polymers, 4D printing enables the production of parts that can change shape and movement.

Research on the design and manufacturing of wings that can change shape is ongoing, with inspiration drawn from nature, particularly bird flight. Folding-wing mechanisms, such as those proposed by Lockheed Martin, allow for changes in the wing’s planform area, resulting in different flight characteristics [14]. However, there are challenges to address, including the complexity of mechanical actuators, increased weight, and the lack of specific dimensions for optimal wing shapes. Another approach involves varying the wing’s camber, and SMAs have been used for this purpose [15]. However, SMAs are dense and require high-temperature stimuli, making them less ideal for UAV wings. The use of biodegradable shape memory polymers could be a potential solution due to their lightweight properties, but further research is needed in this area.

The study explores improvements in 4D printing of biodegradable shape memory polymers for disposable UAVs. Focusing on wing design, it integrates shape memory effect for temperature-triggered changes. Starting with conventional wing methods, it aims to enhance efficiency, flight characteristics, and manufacturing simplicity, aiming to overcome design limitations. The goal is to innovate design techniques that enhance UAV efficiency, eliminate constraints, and simplify manufacturing through 4D printing.

## 2. Methodology

The process of designing a disposable UAV system with shape-changing wings and manufacturing them through 4D printing involves a step-by-step approach. The initial step is to design the wings using traditional UAV design methods, followed by repeating certain design aspects to meet different requirements. Next, the properties necessary for achieving the shape memory effect are determined, leading to the selection of a suitably shaped memory polymer. These steps are introduced in the context of a small, fixed-wing UAV powered by a jet engine, but the research can be extended to other types of UAVs.

### 2.1. Design Considerations

**Figure 1:**
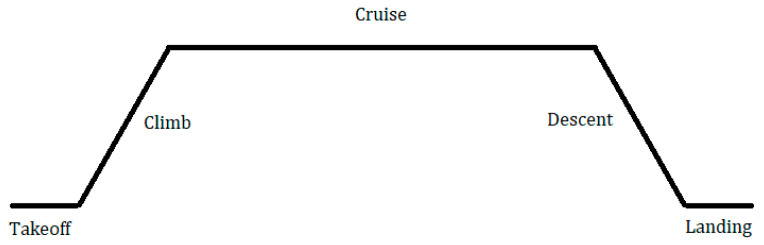
Typical aircraft flight mission.

UAVs are designed to successfully complete each step in their flight missions. As demonstrated in Figure 1, a typical flight mission consists of 5 steps: takeoff, climb, cruise, descent, and landing [16,17]. Takeoff is the horizontal distance the UAV will travel on the ground before it is able to lift off. The climb step is when the UAV rises in altitude until it reaches its operating altitude. At the cruise step, the UAV has reached this altitude and maintains level flight. When the mission is coming to an end, the UAV will begin the descent, which involves the UAV decreasing in altitude until reaching the ground. Once it reaches the ground, it will begin the landing step, in which it again travels vertically on the ground until it comes to a complete stop [16].

The bulk of the entire flight mission is typically the cruise step. All the other steps combined only take up a small percentage of the overall mission compared to the cruise step. Therefore, aircraft design mainly revolves around the flight characteristics during the cruise step [16]. This means that much of the design revolves around only one flight speed and one altitude. UAV and aircraft design additionally revolve around the two main aerodynamic forces seen in flight: lift and drag. Lift is the vertical force needed to lift the UAV off the ground and additionally increase in altitude. Drag is the horizontal force created by the wind opposing the flight of the UAV. The two forces can be calculated using the following equations:

(1) $$L=\frac{1}{2}\rho V^{2}SC_{L}$$

(2) $$D=\frac{1}{2}\rho V^{2}SC_{D}$$

where *ρ* is the air density at a specific altitude, *V* is the flight speed at the altitude, *S* is the wing area, *C_L_* is the lift coefficient, and *C_D_* is the drag coefficient [16,17]. The lift and drag coefficients are nondimensionalized values that are made to simplify the calculations in the design process by using one value that combines the effects of multiple parameters while also eliminating units [18]. Because of this, the lift and drag coefficients are more often used in the design process rather than their associated forces. Additionally, the lift and drag coefficients vary with air density (i.e., with altitude) and speed, so there are many different specific lift and drag coefficients, for example, the zero-lift drag coefficient, which is applied in instances where there is no lift [16,17].

### 2.2. Preliminary Design

In preliminary wing design, initial values are calculated as a starting point, including the UAV’s max takeoff weight, wing area, and engine thrust. Estimations encompass weight components (payload, autopilot, etc.) using statistics, and UAV weight using data. Wing area and thrust are found via five equations linked to performance needs. The first performance requirement that is observed is the stall speed. Stall speed is the minimum speed at which the UAV system will be able to fly while still being able to maintain level flight. The equation associated with the stall speed requirement is

(3) $${\left(\frac{W}{S}\right)}_{V_{S}}=\frac{1}{2}\rho V_{S}{}^{2}C_{L_{max}}$$

where *ρ* is the air density, *V_S_* is the stall speed, and $C_{L_{max}}$ is the maximum lift coefficient [16,17]. 

Once the minimum speed is known, the maximum speed must then be determined as well. Maximum speed is the next performance requirement involved in this process, and its accompanying equation is

(4) $${\left(\frac{T_{SL}}{W}\right)}_{V_{max}}=\rho_{o}V_{max}^{2}C_{D_{o}}\frac{1}{2\left(\frac{W}{S}\right)}+\frac{2K}{\rho \sigma V_{max}^{2}}\left(\frac{W}{S}\right)$$

where *ρ_o_* is the air density at sea level, Vmax is the maximum speed, *C_Do_* is the zero-lift drag coefficient, *σ* is the relative density, and *K* is the induced drag factor [16,17].

The next performance requirement on the list is the maximum rate of climb (*ROC*). The *ROC* is the speed at which an aircraft increases in altitude. In other words, it is the vertical speed of the aircraft. The equation is

(5) $${\left(\frac{T}{W}\right)}_{ROC}=\frac{ROC}{\sqrt{\frac{2}{\rho \sqrt{\frac{C_{D_{o}}}{K}}}\left(\frac{W}{S}\right)}}+\frac{1}{{\left(\frac{L}{D}\right)}_{max}}$$

where (*L/D*)_*max*_ is the lift-to-drag ratio [16,17].

The next requirement, the takeoff run, is the distance that an aircraft must travel before takeoff is successful. This distance is based on how far the aircraft needs to travel horizontally before it can clear a certain obstacle. Generally, this obstacle is imaginary and more of a theoretical idea behind a specified height that the aircraft should be able to reach and exceed for successful takeoff. The associated equation is

(6) $${\left(\frac{T}{W}\right)}_{ROC}=\frac{\mu -\left(\mu +\frac{C_{D_{G}}}{C_{L_{R}}}\right)\left[\exp\left(0.6\rho gC_{D_{G}}S_{TO}\frac{1}{\left(\frac{w}{s}\right)}\right)\right]}{1-\exp\left(0.6\rho gC_{D_{G}}S_{TO}\frac{1}{\left(\frac{w}{s}\right)}\right)}$$

where *μ* is the friction coefficient, *C_DG_* is the ground drag coefficient, *C_LR_* is the lift coefficient at rotation, *g* is the gravitational acceleration, and *S_TO_* is the takeoff run [16,17].

The last performance requirement that is used in this process is the ceiling requirement. The ceiling is defined as the maximum altitude at which an aircraft can safely maintain a straight, level flight There are four different levels of the ceiling which are each defined as the maximum altitude that a specific *ROC* achieves. The four ceilings and their *ROC*s are the absolute ceiling (*ROC* = 0 m/s), service ceiling (*ROC* = 0.5 m/s), cruise ceiling (*ROC* = 1.5 m/s), and combat ceiling (*ROC* = 5 m/s). The last equation is

(7) $${\left(\frac{T}{W}\right)}_{C}=\frac{ROC_{c}}{\sigma_{C}\sqrt{\frac{2}{\rho_{C}\sqrt{\frac{C_{D_{o}}}{K}}}\left(\frac{W}{S}\right)}}+\frac{1}{\sigma_{C}{\left(\frac{L}{D}\right)}_{max}}$$

where *ROC_C_* is the rate of climb at the ceiling, *ρ_C_* is the air density at the ceiling, and *σ_C_* is the relative density at the ceiling [16,17].

**Figure 2:**
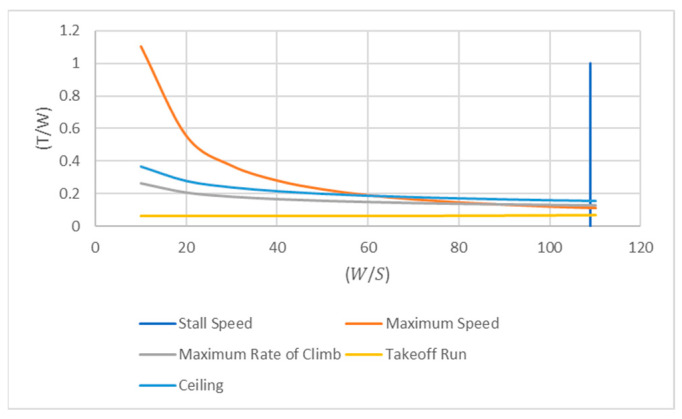
Matching plot example.

When all the values in each equation are known, they can be plugged in to simplify the equations. The five resulting equations must then be graphed simultaneously, as shown in Figure 2. This graph is called a matching plot. The vertical axis of the graph is the thrust loading (*T/W*), and the horizontal axis is the wing loading (*W/S*). The thrust loading is the ratio of the thrust to the weight, which determines how much weight is carried by each unit of thrust. The wing loading is the ratio of the weight to the wing area, which determines the amount of load that is held by each unit area of the wing. From this matching plot, an acceptable region that fully satisfies all the requirements must be chosen. For each equation individually, the regions that satisfy each requirement lie above each curve, except for the stall speed equation. The values that will satisfy the stall speed requirement will all be below the value calculated in this equation. The final acceptable region will be the area of the graph where all five individual regions coincide. Next, a design point must be chosen. The design point will be the lowest point in the chosen region because this will result in the lowest thrust and hence the smallest engine. This point will then determine the thrust and wing loading. The X-value of the design point is the resulting wing loading (*W/S*)_*d*_, and the y-value of the design point is the thrust loading (*T/W*)_*d*_.

After the design point has been selected, the wing loading and thrust loading values will be used to finally calculate the wing area and engine thrust. The associated equations are, respectively,

(8) $$S=\frac{W_{TO}}{{\left(\frac{W}{S}\right)}_{d}}$$

where *W_TO_* is the takeoff weight, and (*W/S*)_*d*_ is the wing loading associated with the design point, and

(9) $$T={\left(\frac{T}{W}\right)}_{d}\times W_{TO}\;$$

where (*T/W*)*_d_* is the thrust loading associated with the design point [16,17]. This completes the preliminary design process.

### 2.3. Wing Design

The wing design process focuses on wing shape, categorized into wing planform area, chord, wingspan, aspect ratio, and airfoil shape. Wing planform area, calculated previously, guides further dimension calculations. Wingspan spans horizontally, while chord represents vertical width. Calculations involve multiplying length and width, where chord can be constant or variable based on wing shape [16,17]. If the wing is rectangular, then the chord is constant. Just as the area of a rectangle is calculated, the wing area can then be calculated using the following equation:

(10) S=b×C

where *b* is the wingspan, and *C* is the chord [16,17].

In cases of trapezoidal wings, where the chord is not consistent, the mean aerodynamic chord (MAC) is employed. MAC is determined by averaging the tip chord (shortest outer chord) and the root chord (longest chord at wing–fuselage connection). This average value replaces the variable chord in the wing area equation for calculations. The last parameter that will be used in this process is the aspect ratio, which is the ratio of the wingspan to the chord:

(11) $$AR=\frac{b}{C}$$

where *AR* is the aspect ratio [16,17]. Generally, a high aspect ratio results in a long, narrow wing, and a low aspect ratio results in a short, broad wing. Like the wing area, the *AR* is determined during preliminary design, so it can also be used when calculating the remaining dimensions.

To calculate the wingspan and chord, the *AR* equation is first observed. By rearranging it to solve for the chord instead, the result is

(12) $$C=\frac{b}{AR}$$

This can then be plugged into the wing area equation:

(13) $$S=\frac{b^{2}}{AR}$$

Because the *AR* and wing area are already known, the equation can be rearranged again to solve for the wingspan:

(14) $$b=\sqrt{S\times AR}$$

Lastly, the wingspan and *AR* can then be plugged in to solve for the chord. Once all the dimensions are known, the cross-sectional shape of the wing must be designed or chosen. This is known as the airfoil shape or simply the airfoil. The airfoil can either be designed or chosen from existing designs. Airfoil design is a very extensive process that requires expertise to accomplish, so a process for choosing an appropriate airfoil will be demonstrated instead. The airfoil selection process will be done by choosing from NACA 5 series airfoils. This is the most logical choice for this process, mainly because of the first digit in the 5-digit airfoil titles. The first digit comes from the design lift coefficient. By multiplying the first digit of a 5-series NACA airfoil by 3/20, the result is then the design lift coefficient. Conversely, the design lift coefficient can also be calculated first and then divided by 3/20 to determine the first digit of the airfoil that should be selected. This is the process that will be used to choose the airfoil.

Airfoil selection, aside from the initial digit, is supported by the Airfoil Tools website. Users can explore a range of airfoil shapes from various sources by inputting known parameters or browsing characteristics on the site. For the NACA 5-series airfoils, *Airfoil Tools* asks the user to input the following parameters:

1. Design coefficient of lift
2. Camber positions and reflex
3. Thickness
4. Number of points

The design coefficient of lift determines the first digit; camber position and reflex affect the second and third digits; thickness influences the fourth and fifth digits. Point quantity aids CAD design (e.g., Solidworks); the chosen airfoil offers points for sketching. Users can input 20 to 200 points, with the website defaulting to 81 points for a smoother sketch. As this design is theoretical and will not be produced, this is not an important value right now, so it will be kept at the default value. If the design is being produced, it is best to choose a high number of points for better accuracy and to avoid any unnecessary “roughness”. The design lift coefficient that will be used will be the ideal lift coefficient of the wing. This can be calculated by using the three following equations [16]:

(15) $$C_{L_{c}}=\frac{2W_{ave}}{\rho V_{C}^{2}S}\;$$

(16) $$C_{L_{C_{W}}}=\frac{C_{L_{c}}}{0.95}$$

(17) $$C_{l_{i}}=\frac{C_{L_{C_{W}}}}{0.9}$$

Equation (15) is the cruise lift coefficient of the UAV. Equation (16) then determines the cruise lift coefficient of the wing only. Lastly, Equation (17) gives the ideal lift coefficient of the wing airfoil [16]. The logic behind the above equations is that while the wing is the main contributor of lift for the UAV, other components contribute as well. In some cases, other components of the UAV can contribute up to 20% of the lift development. The exact contribution varies according to each design, so instead, it can be estimated at this point in the process. The value of 0.95 is assuming a 95% contribution of the lift coming from the wing and 5% from all other components combined. Equation (17) involves a similar thought process, with an estimation of 90% contribution from the airfoil relative to the entire wing [16].

The next required input is the camber position and reflex. The camber can be defined as the curvature seen in the airfoil shape [16,18]. Whether or not the camber is reflexed then depends on the requirements of the UAV and can be decided by the designer. Next, the thickness must be input. Thickness is reflected as a percentage, which comes from the ratio between the thickness and the chord. Generally, a low-speed, high-lift requirement uses a thickness between 15% and 18%, high-speed and low-lift requirements use a thickness between 9% and 12%, and supersonic requirements use a thickness between 3% and 9% [16].

### 2.4. Shape Memory Polymers

Disposable UAV wings utilize heat-responsive shape memory polymers (SMPs) for shape transformation. SMPs shift from a fixed glass state to an elastic form when heated above their glass transition temperature (Tg). The programming process involves reshaping by heating, setting the temporary shape by cooling, and reverting with reheating. SMPs can be trained for various memory effects, including two-way, by selecting appropriate transition temperatures. For UAVs, SMPs with suitable glass temperatures for expected flight conditions are vital. Customizable biodegradable polyurethanes can be created by blending components and adjusting ratios to meet specific needs. Below are a few possible biodegradable SMPs, their glass transition temperature ranges, current applications, and mechanical properties at different temperatures:

- Polycaprolactone (PCL):
  - Glass transition temperature range: −60 °C to −40 °C.
  - Applications: Tissue engineering scaffolds, drug delivery systems, orthopedic devices.
  - Mechanical properties: PCL exhibits good mechanical strength, flexibility, and biocompatibility. Its Young’s modulus ranges from 0.05 to 0.5 GPa.
- Poly(lactic acid) (PLA):
  - Glass transition temperature range: 55 °C to 65 °C.
  - Applications: Biodegradable implants, tissue engineering, drug delivery systems.
  - Mechanical properties: PLA has a tensile strength ranging from 50 to 70 MPa and a Young’s modulus between 2 to 5 GPa, providing moderate strength and stiffness.
- Poly(glycolic acid) (PGA):
  - Glass transition temperature range: 30 °C to 40 °C.
  - Applications: Suture materials, tissue engineering scaffolds, wound healing devices.
  - Mechanical properties: PGA has a high tensile strength, ranging from 40 to 90 MPa, and a relatively low Young’s modulus, typically around 1 GPa.
- Poly(ε-caprolactone-co-lactide) (PCLA):
  - Glass transition temperature range: −25 °C to 5 °C.
  - Applications: Biodegradable stents, tissue engineering, drug delivery systems.
  - Mechanical properties: PCLA exhibits good mechanical strength, with a tensile strength ranging from 15 to 50 MPa and a Young’s modulus around 200 MPa.
- Poly(trimethylene carbonate) (PTMC):
  - Glass transition temperature range: −60 °C to −30 °C.
  - Applications: Vascular stents, tissue engineering, controlled drug-release systems.
  - Mechanical properties: PTMC possesses excellent flexibility and elasticity, with a tensile strength ranging from 5 to 20 MPa and a Young’s modulus around 100 MPa.

## 3. Morphing Wing Designs

The concept’s validation occurs through demonstration. The initial design becomes the permanent wing shape, while adjustments yield temporary designs. Temperatures for shape changes are determined using standard altitudes. After designing, benefits and drawbacks of 4D printed wings are evaluated for flight efficiency and manufacturing.

### 3.1. Initial Design

The initial stage of the design process involves creating a simplified wing design as a starting point for disposable UAV systems. The design will be based on a combination of requirements and estimated values, minimizing overcompensation and proving the effectiveness of a simple design. The wing shape will be rectangular, avoiding tapering. The design is focused on a small disposable UAV system and draws inspiration from the Coyote UAS by Raytheon Technologies, aiming to improve flight characteristics such as range and endurance. The Coyote UAS is chosen as a reference due to its multi-mission capabilities and extreme characteristics compared to other small UAV systems. Statistical data from [16] will inform the estimated values chosen for the design. According to statistical data, small UAVs generally can travel up to 5000 feet in altitude, 100 km in range, and for up to 2 h. The specifications of the Coyote provided by the manufacturer are [19]

1. Weight: 13 lb
2. Length: 36 inches (0.91 m)
3. Wingspan: 58 inches (1.5 m)
4. Altitude: 30,000 ft (9100 m)
5. Cruising speed: 55 knots (28 m/s)
6. Maximum speed: 70 knots (36 m/s)
7. Range: 50 miles (93 km)
8. Endurance: 1 h

Typically, small UAVs (5 to 55 lb) fly at a cruising altitude between 1000 and 5000 ft, with a range of 10 to 100 km (about 6.2 to 62 mi) and an endurance of 0.5 to 2 h [17]. When comparing these characteristics to the statistical values, it is evident that the Coyote is capable of flying much higher than other small UAV systems. It also flies at relatively high speeds for its size; however, the range and endurance of the Coyote fall within the provided range. In fact, while the range is nearly maximized according to the statistical estimation, the endurance is quite low in comparison. This will be a good example to use as the basis of this design process because it has extreme capabilities but can also use improvements. The goal is to design wings for a disposable UAV system that is similar in size and performance and then explore possible ways to improve it by utilizing the shape memory effect. By doing so, the need for high-lift devices is eliminated, thus simplifying both the design and manufacturing processes.

Before beginning the design process, some initial requirements must be established. While the goal is to design wings for disposable UAV systems similar to the Coyote, the exact values will not be used. Instead, similar goal values will be chosen, some of which will result in easier calculations. The requirements will be

1. Weight: 10 lb
2. Cruising speed: 25 m/s
3. Cruising altitude: 5000 ft

The minimum requirements for the design process have been established, with options for estimating unknown values. The cruising speed is chosen to enable cleaner calculations, while the weight and cruising altitude are based on statistical data. The cruising altitude is initially set to maximize the potential of the UAV, and variations in speed will be considered. The weight falls within the middle range of statistical data, making it more manageable. With the minimum requirements defined, the design process can proceed, starting with the preliminary design phase. This phase involves estimating the maximum takeoff weight, wing planform area and required thrust based on the given requirements. The calculations rely on statistical values and additional equations provided by Sadraey (2013, 2020) [16,17].

The first performance requirement and its associated characteristics that will be analyzed is the stall speed. Stall speed is the minimum speed an aircraft can fly at before failing; if the aircraft flies below the stall speed, it will not be able to produce lift and, therefore, cannot remain airborne [16,17]. Additionally, by minimizing the stall speed, the UAV system can fly at very low speeds if its missions call for it, since hovering is not an option for fixed-wing UAV systems. Based on statistical data about other small UAV systems in Table 1, the stall speed used will be 10 m/s. The associated maximum lift coefficient will be 1.8. For the air density, it is suggested to use the air density at sea level to maximize the estimation. However, this may not provide the most accurate result. Since the stall speed is the lowest speed at which level flight is maintained, the “level flight” is what should be considered. If an aircraft is performing level flight, this means that it is flying at a constant altitude. Generally, level flight implies cruising, so the cruising altitude will be observed, and its associated air density will be used in the equation. The cruising altitude was said to be 5000 ft, and the air density at this altitude is 1.0555 kg/m^3^. Now, all the values have been determined and can be plugged into the following equation:

(18) $${\left(\frac{W}{S}\right)}_{V_{S}}=\frac{1}{2}\rho V_{S}{}^{2}C_{L_{max}}=\frac{1}{2}\left(1.0555\frac{\mathrm{kg}}{m^{3}}\right){\left(10\frac{m}{s}\right)}^{2}\left(1.8\right)\;=94.995\frac{N}{m^{2}}$$

The next performance requirement is the maximum speed. Like the stall speed, the maximum speed is usually decided in advance by either the customer or the designer, but if it has not been decided, then it can be estimated using the cruising speed. Generally, the maximum speed is 20% to 30% higher than the cruising speed, so the maximum speed here will be

(19) $$V_{max}=1.2V_{C}=1.2\left(25\frac{m}{s}\right)=30\frac{m}{s}$$

where *V_C_* is the cruising speed [13]. The initial density will be the density at the takeoff altitude. For very large aircraft, takeoff usually begins on the ground (i.e., at sea level), but small UAVs can take off from various places. To keep it simple, the takeoff altitude will be assumed to be sea level; thus, the air density that will be used is 1.225 kg/m^3^. Additionally, the air density at the given flight altitude is needed as well. The altitude that is used here is the altitude that the UAV system will be flying at when flying at maximum speed. Because this value was also not predetermined, it will be estimated the same way that the maximum speed was estimated, since speed tends to increase with altitude. The resulting altitude is then 6000 feet (or 1829 m), and the air density at this altitude is about 1.0239 kg/m^3^. Now that both air densities are known, the relative density can be calculated. The relative density is the ratio of the air density at the given altitude to the air density at sea level. The result is

(20) $$\sigma =\frac{\rho_{alt}}{\rho_{SL}}=\frac{1.0239\frac{kg}{m^{3}}}{1.225\frac{kg}{m^{3}}}=0.8358$$

where *ρ_alt_* is the air density at the specified altitude, and *ρ_SL_* is the air density at sea level [16]. The next calculation will be for the induced drag factor. First, the Oswald efficiency factor and aspect ratio must be chosen. The Oswald span efficiency factor is generally between 0.7 and 0.95 [16], so 0.7 will be used. The aspect ratio will be 9, estimated from Table 2. The induced drag factor is then

(21) $$K=\frac{1}{\pi \times e\times AR}=\frac{1}{\pi \left(0.7\right)\left(9\right)}=0.0505$$

where *e* is the Oswald efficiency factor. Lastly, the zero-lift drag coefficient will be estimated from statistical data. The estimation is conducted based on the typical range for microlight aircraft, as seen in Table 3. To avoid overestimation, the lower value is chosen, so the zero-lift drag coefficient will be 0.02. The obtained values can now be plugged into the original equation:

(22) $${\left(\frac{T_{SL}}{W}\right)}_{V_{max}}=\rho_{o}V_{max}^{2}C_{D_{o}}\frac{1}{2\left(\frac{W}{S}\right)}+\frac{2K}{\rho \sigma V_{max}^{2}}=\left(1.225\right){\left(30\right)}^{2}\left(0.02\right)\frac{1}{2\left(\frac{W}{S}\right)}+\frac{2\left(0.0505\right)}{\left(1.0239\right)\left(\frac{1.0239}{1.225}\right){\left(30\right)}^{2}}\left(\frac{W}{S}\right)\;=\frac{11.025}{\frac{W}{S}}+0.0001311\;\left(\frac{W}{S}\right)$$

**Figure 3:**
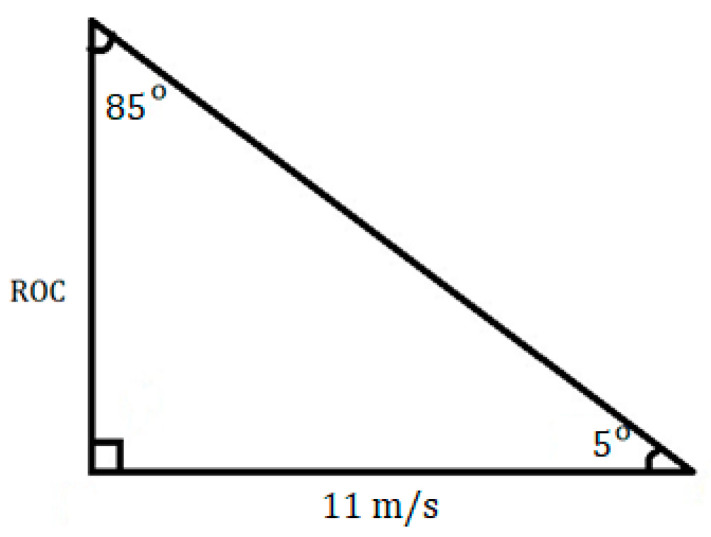
Rate of climb trigonometry.

The next performance requirement is the maximum rate of climb (*ROC*). The *ROC* can be seen as the vertical speed of an aircraft. With this in mind, the maximum *ROC* can be estimated using the horizontal speed and the angle of incidence of the wings, which is the angle between the chord of the wing and the centerline of the fuselage [16]. The *ROC* is maximized at sea level, so the horizontal speed that will be used to estimate the maximum *ROC* is the takeoff speed [16]. The takeoff speed can then be estimated from the stall speed. Typically, the takeoff speed is 10% to 30% higher than the stall speed, so it will be estimated as follows:

(23) $$V_{TO}=1.1\;V_{S}=1.1\left(10\frac{m}{s}\right)=11\;m/s$$

where *V_TO_* is the takeoff speed [16]. Typical values for the angle of incidence are generally low, so the angle that will be used will be a 5° angle. With these two values, the vertical speed can be calculated using simple trigonometry. These values can all be envisioned on a triangle, as shown in Figure 3.

Through basic trigonometry, the *ROC* can be determined by

(24) $$\tan\left(5{}^{\circ}\right)=\frac{ROC}{11\frac{m}{s}}$$

Then, the identity can be rearranged to solve for the ROC, rounding to the nearest m/s:

(25) $$ROC=\left(11\frac{m}{s}\right)\tan\left(5{}^{\circ}\right)=1\frac{m}{s}$$

This gives a good estimation for the maximum rate of climb based on other estimations and requirements, so this process can be used when the *ROC* is not specified. Lastly, the lift-to-drag ratio will be estimated to be 15 from Table 4. Now that all values have been chosen, they can be plugged into the following equation:

(26) $${\left(\frac{T}{W}\right)}_{ROC}=\frac{ROC}{\sqrt{\frac{2}{\rho \sqrt{\frac{C_{D_{o}}}{K}}}\left(\frac{W}{S}\right)}\;}+\frac{1}{{\left(\frac{L}{D}\right)}_{max}}=\frac{1}{\sqrt{\frac{2}{1.225\sqrt{\frac{0.02}{0.0505}}}\left(\frac{W}{S}\right)}\;}+\frac{1}{15}\;=\frac{0.6209}{\sqrt{\left(\frac{W}{S}\right)}}+\frac{1}{15}$$

The take-off run will be the next performance requirement. This step is a bit more complex, with more values and equations to solve to obtain all the necessary parameters. The take-off run distance and the friction coefficient depend on where the UAV system is taking off from, as well as its performance. The take-off run will be considered the distance needed for the UAV to clear a 15 m obstacle [16]. This distance will be estimated using the maximum rate of climb since the *ROC* is maximized at sea level. The maximum ROC was determined to be about 1 m/s; thus, it will take about 15 s to rise to 15 m. Then, the takeoff speed can be utilized to determine what horizontal distance will be traveled in the given amount of time. Using the takeoff speed of 11 m/s, in 15 s the UAV will travel 165 m horizontally, and this will be the takeoff run.

Since UAV systems are usually designed for specific missions, the surface that it is taking off from is usually known or assumed. Because this design is theoretical, and since the goal is to design a multi-purpose UAV, it is difficult to know what kind of surface the UAV system will travel on because it can vary. The best way to estimate the friction coefficient will be to pick a value somewhere in the middle of the overall range. Based on values from Table 5, friction coefficients all range from 0.02 to 0.3. However, most fall in the range of 0.02 to 0.07, so it will be best to fall somewhere in between these values. The friction coefficient will then be 0.05, as this value qualifies for multiple surface types. The next two parameters are air density and acceleration due to gravity. These are simple, as no calculations need to be conducted. Since the UAV system is not yet airborne during this part of the mission, sea-level values can again be used, so the air density is 1.225 kg/m^3^, and the acceleration due to gravity is 9.81 m/s^2^. Now that the simple parameters have been determined, next will be the collection of steps needed to calculate the associated drag and lift coefficients. The lift coefficient at rotation is relatively simple, as it is an estimation from the maximum lift coefficient, which has also been estimated in a previous step. It will be calculated to be the following:

(27) $$C_{L_{R}}=\frac{C_{L_{max}}}{{\left(1.1\right)}^{2}}=\frac{1.8}{{\left(1.1\right)}^{2}}=1.4876$$

The drag coefficient will take a few more equations to solve. The order of the equations is as follows:

(28) $$C_{L_{TO}}=C_{L_{C}}+\Delta C_{L_{flap\_TO}}$$

where *C_DoTO_* is the zero-lift drag coefficient at takeoff, *C_DoLG_* is the zero-lift drag coefficient of the landing gear, and *C_DoHLD_TO_* is the zero-lift drag coefficient of the HLD at take-off [16];

(29) $$C_{L_{TO}}=C_{L_{C}}+\Delta C_{L_{flap\_TO}}$$

where *C_LTO_* is the lift coefficient at takeoff, *C_LC_* is the cruise lift coefficient, and *C_Lflap_TO_* is the lift coefficient of the flap (if one will exist) at take-off [16];

(30) $$C_{D_{TO}}=C_{D_{oTO}}+KC_{L_{TO}}^{2}$$

where *C_DTO_* is the drag coefficient at takeoff [16];

(31) $$C_{D_{G}}=C_{D_{TO}}-\mu C_{L_{TO}}$$

where *C_DG_* is the ground drag coefficient [16].

The Equations (30) and (31) can be reduced as well, as HLDs and flaps are not being considered, so *C_DoHLD_TO_* and *∆C_LflapTO_* will be zero. Then, *C_LC_* will be 0.3, as this is the value that it generally falls on in other designs. The results for them are then

(32) $$C_{D_{oTO}}=C_{D_{o}}+C_{D_{oLG}}+C_{D_{oHLD\_TO}}=0.02+0.006=0.026$$

(33) $$C_{L_{TO}}=C_{L_{C}}+\Delta C_{L_{flapTO}}=0.3$$

Now, these two values can be plugged into Equation (30):

(34) $$C_{D_{TO}}=C_{D_{oTO}}+KC_{L_{TO}}^{2}=0.026+\left(0.0505\right){\left(0.3\right)}^{2}=0.0305$$

Finally, the ground drag coefficient can be solved using the following equation:

(35) $$C_{D_{G}}=C_{D_{TO}}-\mu C_{L_{TO}}=0.0305+\left(0.05\right)\left(0.3\right)=0.0320$$

All parameters have now been determined, so plugging them into the original equation results in

(36) $${\left(\frac{T}{W}\right)}_{ROC}=\frac{\mu -\left(\mu +\frac{C_{D_{G}}}{C_{L_{R}}}\right)\left[\exp\left(0.6\rho gC_{D_{G}}S_{TO}\frac{1}{\frac{w}{s}}\right)\right]}{1-\exp\left(0.6\rho gC_{D_{G}}S_{TO}\frac{1}{\frac{w}{s}}\right)}=\frac{0.05-\left(0.05+\frac{0.0320}{1.4876}\right)\left[\exp\left(0.6\left(1.225\right)\left(9.81\right)\left(0.0320\right)\left(165\right)\frac{1}{\left(\frac{w}{s}\right)}\right)\right]}{1-\exp\left(0.6\left(1.225\right)\left(9.81\right)\left(0.0320\right)\left(165\right)\frac{1}{\left(\frac{w}{s}\right)}\right)}\;=\frac{0.05-0.0715\left[\exp\left(38.0706\left(\frac{1}{\frac{W}{S}}\right)\right)\right]}{1-\exp\left(38.0706\left(\frac{1}{\frac{W}{S}}\right)\right)}$$

Finally, the last requirement is the ceiling. Because much of the flight mission for a small UAV system involves cruising flight, the cruise ceiling will be used. Because the cruise ceiling is being used, the associated ROC is 1.5 m/s. It also means that the ceiling is equal to the cruising altitude, which is 5000 ft or 1524 m. The air density at 5000 ft is about 1.0555 kg/m^3^, and the resulting relative density is 0.8616. Plugging everything into the last equation gives the following:

(37) $$\begin{array}{c} {\left(\frac{T}{W}\right)}_{C}=\frac{ROC_{c}}{\sigma_{C}\sqrt{\frac{2}{\rho_{C}\sqrt{\frac{C_{D_{o}}}{K}}}\left(\frac{W}{S}\right)}}+\frac{1}{\sigma_{C}{\left(\frac{L}{D}\right)}_{max}}=\frac{1.5}{\left(0.8616\right)\sqrt{\frac{2}{\left(1.0555\right)\sqrt{\frac{0.02}{0.0505}}}\left(\frac{W}{S}\right)}}+\frac{1}{\left(0.8616\right)\left(15\right)}\\ \;\;\;\;\;=\frac{1.0033}{\sqrt{\left(\frac{W}{S}\right)}}+\frac{1}{12.924} \end{array}$$

**Figure 4:**
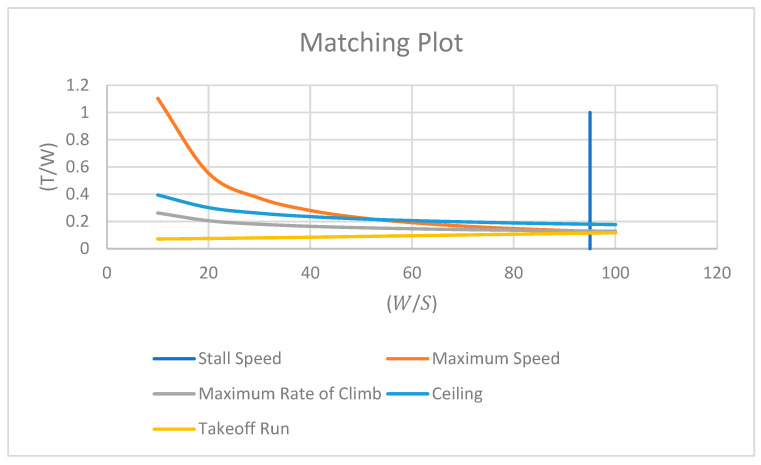
Matching plot containing the resulting 5 curves from preliminary design calculations.

Now that all the equations have been simplified using all the necessary parameters, they can be graphed on a matching plot. All the equations will be plotted together, and the design point will be marked. As explained in the Section 2.2, the acceptable regions are above each curve, except the stall speed equation, which has an acceptable region to the left of the line. The acceptable region for all equations combined is then the region above the ceiling curve and to the left of the stall speed line, as seen in Figure 4. When choosing the design point, the point must be chosen that falls in this region, but it is best to choose the point with the lowest thrust because this will result in a smaller engine. This happens at the intersection point of the ceiling curve and the stall speed line. The values of the point are

(38) $${\left(\frac{W}{S}\right)}_{d}=94.995$$

and

(39) $${\left(\frac{T}{W}\right)}_{d}=0.1803$$

Using these values, the wing area and thrust can be calculated:

(40) $$S=\frac{W_{TO}}{{\left(\frac{W}{S}\right)}_{d}}=\frac{44.5\;N}{94.995}=0.4684\;m^{2}\;$$

and

(41) $$T={\left(\frac{T}{W}\right)}_{d}\times W_{TO}=0.1803\times 44.5\;N=8.0234\;N$$

The next step in the design process will be wing design. This step will determine the wingspan, chord, and airfoil. Using the wing area and aspect ratio, the wingspan is

(42) $$b=\sqrt{S\times AR}=\sqrt{\left(0.4829m^{2}\right)\left(9\right)}=2.0847\;m$$

Next, the chord length can be calculated:

(43) $$C=\frac{S}{b}=\frac{0.4829\;m^{2}}{2.0847\;m}=0.2316\;m$$

Now that all the necessary dimensions of the wings have been determined, the next step is to decide on the shape of the wing. This is also known as the airfoil shape, airfoil section, or simply airfoil of the wing. At this point in the design process, the wing has been designed according to a cruising altitude of 5000 ft and the associated air characteristics. While the plan is to explore possibilities of training the wings to change shape in order to adjust to changing altitudes and/or air characteristics, the process will first start with choosing an airfoil that is useful for cruising flight at 5000 ft.

First, the design lift coefficient must be calculated by using the three equations that have been previously introduced. By plugging in all the parameters, which have already been determined and used multiple times, the results are as follows:

(44) $$C_{L_{c}}=\frac{2W_{ave}}{\rho V_{C}^{2}S}=\frac{2\left(44.5\;N\right)}{\left(1.0555\frac{kg}{m^{3}}\right){\left(25\frac{m}{s}\right)}^{2}\left(0.4684\;m^{2}\right)}=0.2880$$

(45) $$C_{L_{C_{W}}}=\frac{C_{L_{c}}}{0.95}=\frac{0.2880}{0.95}=0.3032$$

(46) $$C_{l_{i}}=\frac{C_{L_{C_{W}}}}{0.9}=\frac{0.3032}{0.9}=0.3369$$

Then, by dividing this value by 3/20, the result is about 2.2. This does not work, as a whole number is needed. This means the first digit will either be rounded down to 2 or rounded up to 3. Normally, when inputting values into the Airfoil Tools, if the inputs do not provide an exact value for the first digit, then the result tends to be rounded down. In this process, however, it is necessary to round up instead. This will be carried out to ensure that the airfoil choice will be able to produce enough lift to reach the desired altitude. If the design lift coefficient is rounded down, then the airfoil may not be able to produce the lift needed to reach the 5000 ft altitude. By using this method, the first digit will then be 3. If needed, the exact altitude associated with this digit and lift coefficient can be calculated, but for now, it will be assumed that 5000 ft is still a useful value. The first digit is now known, but all values must still be inputted into the website to accurately determine the airfoil selection. This means that the design lift coefficient associated with digit 2 must still be known. By multiplying 3 by 3/20, the resulting design lift coefficient is then 0.45. This will be the value to input on the website.

The next input is the camber position and reflex. The camber position is reflected as a percentage relative to the chord. It depicts where the camber will be positioned on the chord, starting from the leading edge [18]. For example, if the chord is 5 cm and there is a 20% camber, this means that the camber will be located 1 cm away from the leading edge since 1 cm is 20% of 5 cm. For this design process, it is best to pick the highest possible percentage because the shape change will be easier to program if the camber is further away from the leading edge. Along with this value, the user must also decide if the camber will be standard or reflexed. A standard camber curves downward, while a reflexed camber curves upward. Reflexed cambers are typically used in the design of aircraft that do not have tails [16]. While it seems easier to choose a reflexed camber to avoid designing and producing tails, they have some disadvantages that may make this choice unsuitable. The main disadvantage is that the reflexed camber is much more sensitive to changes in Reynolds number. Reynolds number changes as velocity, altitude, and/or air density change, or even as the wing area changes, if the design allows for such a concept. Because any of these things can change at any moment, it makes using a reflexed camber a potential risk. To keep this design simple, a standard camber will be considered. The standard choices offered by *Airfoil Tools* are the following:

1. 5% standard (10)
2. 10% standard (20)
3. 15% standard (30)
4. 20% standard (40)
5. 25% standard (50)

The last selection that will be made will be the thickness of the wing and airfoil. For this design, the purpose is to enhance the lift and flight of the UAV rather than making a very high-speed UAV, so the thickness should be chosen based on this. The thickness range for low-speed, high-lift wings is 15% to 18% [16]. While increasing the thickness contributes to the lift, it also means more material, a longer production time, and thus higher cost. Because of this, the thickness will be minimized to 15%.

To summarize, the final selections are as follows:

1. Design coefficient of lift: 0.45
2. Camber position and reflex: 25% standard
3. Thickness: 15%
4. Number of points: 81

The resulting airfoil from these selections is the NACA 35015. As demonstrated in this entire design process, this is an ideal airfoil choice for a UAV system that will be cruising at an altitude of 5000 ft. The resulting wing is simply the initial design. The next step is to determine under what conditions the wing should change shape and what shape it should change to or from.

### 3.2. Alternative Airfoils

The airfoil choice for shape-changing hinges on the desired lift coefficient. Varying the design lift coefficients dictates matching airfoils with constant thickness and camber. Weight stability is assumed, focusing instead on air density, speed, and wing area variations. Using diverse altitudes, including above and below the original, calculations showcase lift coefficient impacts. Ranging from sea level to 1000 ft, then incrementing in 5000 ft steps, different altitudes drive these calculations.

As seen in Table 6, with the determined UAV system weight and wing area, the design lift coefficient greatly varies with altitude. Because the maximum possible design lift coefficient is 1, the highest altitude in regard to the table values would be 35,000 ft, although the exact highest cruising altitude would be somewhere between 35,000 and 40,000 ft. While these calculations successfully demonstrate the variety of potential cruising altitudes and design lift coefficients, the values are not exactly useful since the design lift coefficients will be rounded down when input into *Airfoil Tools*.

Because of this, the next logical idea would be to instead determine what altitudes are associated with the exact design lift coefficients that will be used. To recalculate the associated altitudes, we will first rearrange the ideal lift coefficient equation to solve for air density instead. Because there are actually three equations involved as well, the new calculation process will be a reversal of the original process, meaning the last step will become the first step. The calculation steps then become

(47) $$C_{L_{C_{W}}}=0.9C_{l_{i}}$$

(48) $$C_{L_{c}}=0.95C_{L_{C_{W}}}$$

(49) $$\rho =\frac{2W_{ave}}{C_{L_{c}}V_{C}^{2}S}$$

**Figure 5:**
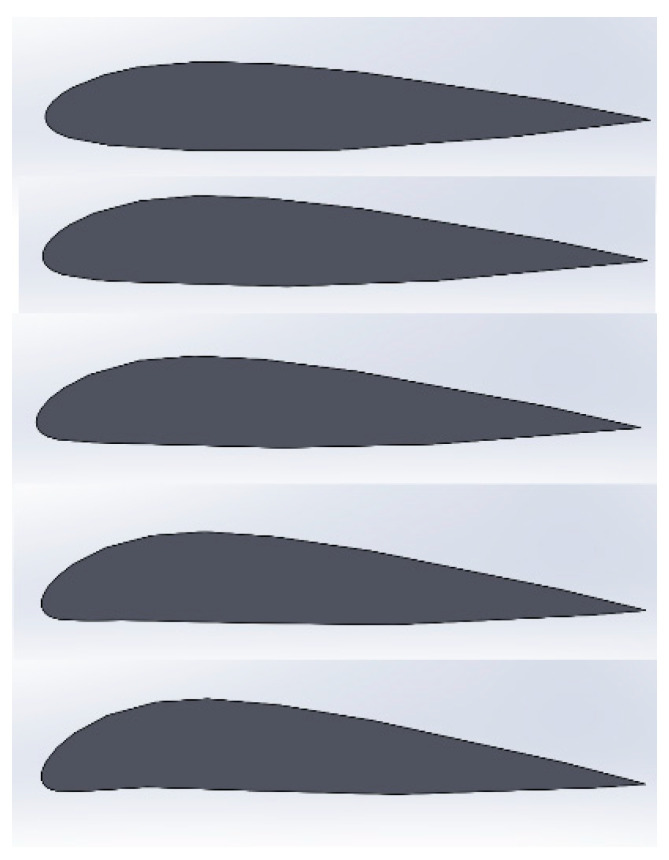
NACA 25015, 35015, 45015, 55015, and 65015 (from top to bottom) plotted on Solidworks.

Using this new calculation process, each design lift coefficient from the table can be plugged in, as well as all the other values needed, and each resulting air density can then be calculated. This will yield the results shown in Table 7. With these results, the options can then be narrowed down. Because the air density at sea level is 1.225 kg/m^3^, the design lift coefficients that are associated with air densities above this value can be eliminated. Additionally, the airfoil of the original wing was chosen using 0.45 as the design lift coefficient. The remaining options for the design lift coefficient are now 0.3, 0.6, 0.75, and 0.9. This means that the airfoil options are then NACA 25015, 45015, 55015, and 65015, as shown in Figure 5. From here, the designer can choose which airfoil the wing will change into and at what point the shape change will happen, or the designer can try to employ a multi-shape memory effect to use two, three, or even all four of the airfoils. Before continuing, it must be noted that because each of these altitudes defines the exact altitude for each design lift coefficient, they should be considered the maximum altitude associated with each design lift coefficient. This means that at each of these altitudes, the shape change that should occur should be to the airfoil associated with the next design lift coefficient rather than the design lift coefficient associated with the altitude. This is carried out to ensure that enough lift is produced to reach each of the altitudes. The range of altitudes at which each airfoil will be used is listed in Table 8.

The next step is to determine the temperature associated with each design lift coefficient and, thus, each airfoil shape. This will be done to determine the needed transition temperatures for the material to be able to change shape. While specific air properties would normally be determined through calculations and/or testing procedures, we will continue to assume standard conditions for the sake of demonstrating the design process. Again, while the air condition is not always standard, the properties can always be determined. This can be included in the process for future research and development and completion of the design.

In addition to the temperature required for shape memory training, a force must be applied to the wings to push and hold them into their new shape. The amount of force applied during the training process should match the force that the UAV system will experience during flight. For fluids, the force can be calculated as the amount of pressure applied to a certain area or

(50) F=PA

Because the fluid (i.e., air) is moving, the pressure used in this equation would be the dynamic pressure rather than the barometric pressure, which is static pressure. The dynamic pressure can be calculated with the air density and velocity [18]:

(51) $$Q=\frac{1}{2}\rho V^{2}$$

The dynamic pressure can then be calculated for each altitude using each of the respective air densities. Table 9 provides all the combined results for the design lift coefficients, altitudes, air densities, temperatures, dynamic pressures, and forces. The altitude for each design lift coefficient is the minimum altitude in each range. In this study, the concept of training the disposable UAV wings to change into multiple shapes is highly desirable. As demonstrated, for the most efficient flight, multiple different airfoil shapes can be employed at multiple different altitudes. The temperatures and forces necessary to program these shape changes have already been determined, so to explore the possibility of training the wing to change to and from all the chosen shapes, the material choice must be determined by the glass transition temperature. The material must also be able to withstand forces greater than those determined by the associated dynamic pressures.

In order to explore the possibility of training the wings to change to and from all five airfoil shapes, a material must be found and chosen that meets the glass transition temperature requirements. The temperatures needed for each of the airfoils range from 15 °C to −42.6 °C. This means that the material used to produce the disposable UAV wings would have to be one with a minimum glass transition temperature less than or equal to −42.6 °C. Once this material is chosen or created, the shape memory effect can be programmed into the wings to alter the shape with changes in altitude.

### 3.3. Alternative Wing Areas

The wing area forms the core of this shape-changing approach, replacing diverse airfoils with recalculations for various altitudes. By repeating design steps using altered altitudes and densities, distinct wing areas emerge, demonstrated using the original design at 5000 ft and recalculating for other altitudes, and considered lower for climbing and higher for potential gain, or both for two-way shape memory effect. Three of five equations change with altitude, requiring recalculation, then adjusting the plot and selecting a new design point to compute the updated wing area.

The process will demonstrate the use of a lower altitude for the shape-changing mechanism. The chosen altitude can either be an alternative cruising altitude for low-altitude flight or an intermediate altitude between sea level and 5000 feet to enhance climbing flight. To adhere to FAA regulations, the process will be shown using an alternative cruising altitude of 400 feet, as UAVs under 55 pounds are limited to that altitude for certain operations conducted above people. By designing the UAV for optimum flight at 400 feet, it can still be utilized for operations above people while remaining useful for missions in non-populated areas that require higher altitude flights, such as forest monitoring or military use.

We refer again to the three equations that are needed:

(52) $${\left(\frac{W}{S}\right)}_{V_{S}}=\frac{1}{2}\rho V_{S}{}^{2}C_{L_{max}}$$

(53) $${\left(\frac{T_{SL}}{W}\right)}_{V_{max}}=\rho_{o}V_{max}^{2}C_{D_{o}}\frac{1}{2\left(\frac{W}{S}\right)}+\frac{2K}{\rho \sigma V_{max}^{2}}\left(\frac{W}{S}\right)$$

(54) $${\left(\frac{T}{W}\right)}_{C}=\frac{ROC_{c}}{\sigma_{C}\sqrt{\frac{2}{\rho_{C}\sqrt{\frac{C_{D_{o}}}{K}}}\left(\frac{W}{S}\right)}}+\frac{1}{\sigma_{C}{\left(\frac{L}{D}\right)}_{max}}$$

First, the stall speed equation will be recalculated. The stall speed and maximum lift coefficient will remain unchanged, and the air density will now be different. The cruising altitude has been reset to 400 ft, and the air density at this altitude is 1.2107 kg/m^3^. Plugging in the new air density,

(55) $${\left(\frac{W}{S}\right)}_{V_{S}}=\frac{1}{2}\rho V_{S}{}^{2}C_{L_{max}}=\frac{1}{2}\left(1.2107\frac{kg}{m^{3}}\right){\left(10\frac{m}{s}\right)}^{2}\left(1.8\right)=108.963\;N$$

Next, the maximum speed equation will be recalculated. The only parameters that have changed are the air density at the maximum speed altitude and the relative density. The altitude where the maximum speed will occur will be estimated as previously demonstrated. The resulting altitude is 480 ft. To simplify the calculations and make it easier to determine the air density, this number will be rounded up to 500 ft, which will be much easier to work with. The air density at 500 ft is 1.2072 kg/m^3^, and the resulting relative density is 0.9855. The new equation is now

$${\left(\frac{T_{SL}}{W}\right)}_{V_{max}}=\rho_{o}V_{max}^{2}C_{D_{o}}\frac{1}{2\left(\frac{W}{S}\right)}+\frac{2K}{\rho \sigma V_{max}^{2}}\left(\frac{W}{S}\right)$$

(56) $$=\left(1.225\frac{\mathrm{kg}}{m^{3}}\right){\left(30\frac{m}{s}\right)}^{2}\left(0.02\right)\frac{1}{2\left(\frac{W}{S}\right)}+\frac{2\left(0.0505\right)}{\left(1.2072\frac{kg}{m^{3}}\right)\left(0.9855\right){\left(30\frac{m}{s}\right)}^{2}}\left(\frac{W}{S}\right)=\;\frac{11.025}{\left(\frac{W}{S}\right)}+0.00009433\left(\frac{W}{S}\right)$$

Next, the ceiling equation will be recalculated. The only parameters that are changing are the air density at the ceiling altitude and the relative air density. Again, the ceiling used here will be the cruising ceiling; thus, the cruising altitude is used. The air density at 400 ft is 1.2107 kg/m^3^, resulting in a relative density of 0.9883. The new equation is

(57) $$\begin{array}{ll} {\left(\frac{T}{W}\right)}_{C} & =\frac{ROC_{c}}{\sigma_{C}\sqrt{\frac{2}{\rho_{C}\sqrt{\frac{C_{D_{o}}}{K}}}\left(\frac{W}{S}\right)}}+\frac{1}{\sigma_{C}{\left(\frac{L}{D}\right)}_{max}}\\ & =\frac{\left(1.5\frac{m}{s}\right)}{\left(0.9883\right)\sqrt{\frac{2}{\left(1.2107\frac{kg}{m^{3}}\right)\sqrt{\frac{\left(0.02\right)}{\left(0.0505\right)}}}\left(\frac{W}{S}\right)}}\\ & +\frac{1}{\left(0.9883\right)\left(15\right)}=\frac{0.9368}{\sqrt{\left(\frac{W}{S}\right)\;}}+\frac{1}{14.8245} \end{array}$$

As in the previous wing design, the design point is the intersection point between the ceiling and stall speed. The wing area is calculated from the X-value of the design point, so this is all that will be considered. The wing loading (X-value) is then

(58) $${\left(\frac{W}{S}\right)}_{d}=108.963\;N$$

The resulting wing area can then be calculated:

(59) $$S=\frac{W_{TO}}{{\left(\frac{W}{S}\right)}_{d}}=\frac{44.5\;N}{108.963}=0.4084\;m^{2}$$

The next step involves redoing the process using altitudes above 5000 ft. The initial goal was to design wings that can adjust to altitude changes and potentially reach altitudes as high as 30,000 ft or even 35,000 ft. To achieve this, the wing area needed for cruising flight at each altitude will be calculated, considering altitudes between 5000 and 35,000 ft in 5000 ft increments. By determining the maximum altitude corresponding to each cruising altitude and the air densities for all altitudes, the relative density can be calculated, leading to the determination of wing areas. Since the aspect ratio remains constant, changes in area will result in changes in wingspan or chord length. Achieving changes in chord length may not be ideal, so focusing on changes in wingspan by folding the wings upward or downward is a preferable method for shape changing. The change in the wingspan will determine where the wing will be folded. The wingspan results have also been provided in Table 10.

### 3.4. Endurance and Range

As the entire potential design processes have been laid out and multiple possible designs provided, the results of employing these types of designs will be analyzed. The goal of these designs is to improve many aspects of both the design process and the actual use of the UAV. To prove that these designs are an improvement over the Coyote, an estimation of the range and endurance will be calculated based on the power required for each shape.

The first step in determining the efficiency of these designs is to calculate the amount of power used throughout the different shape changes. As the flight characteristics of the UAV will change with altitude, the amount of power needed to maintain the cruising flight will change as well. In this study, the design of small UAV systems is considered, and small UAV systems generally tend to be battery powered. To analyze the effects of the shape-changing wings on the efficiency of the UAV flight, a few calculations must be carried out that relate the characteristics of the battery in use and the flight characteristics, which are all provided by Sadraey (2020) [17].

Power can be calculated using thrust and velocity. The general equation is

(60) P=TV

If the UAV is cruising, then the thrust is equal to the drag [16,17], so the following equation can also be used:

(61) P=DV

If drag is being used to calculate the power, then the drag must first be calculated using Equation (2). To calculate the drag force, the drag coefficient must first be calculated accordingly [16]:

(62) $$C_{D}=C_{D_{o}}+\frac{C_{L}}{\pi \times AR\times e}$$

Lastly, the lift coefficient is calculated by rearranging Equation (1) and substituting lift with weight, as they are equal during cruising flight [16,17]:

(63) $$C_{L}=\frac{2W}{\rho \times S\times V^{2}}$$

To determine the power needed to fly at each altitude using the airfoil-changing designs, the process will be to calculate the lift coefficient, then the drag coefficient, then the drag force, and finally the power. Table 11 provides all the results related to each airfoil. The calculations were conducted using the highest altitude in each range.

With this information, the range and endurance can then be calculated for each altitude. This can be carried out by observing the power that will be used, the energy density, and the mass of the battery [17]:

(64) $$m_{B}=\frac{Pt}{E_{D}}$$

This equation is usually used to calculate the battery mass using power, time, and energy density. It can then be rearranged to solve for time:

(65) $$t=\frac{m_{B}E_{D}}{P}$$

Lastly, the range can be calculated from the endurance [16,17]:

(66) $$t_{C}=\frac{R}{V_{C}}$$

Rearranging the equation to solve for the range gives the following:

(67) $$R=t_{C}V_{C}$$

The cruising flight range is then calculated. For overall mission time, the cruising endurance is estimated based on its percentage. Typically, most of the mission involves cruising, minimizing differences. Comparatively, power usage is computed for Coyote using the above equations, making assumptions about its unclear power source. The same assumptions are used for the smart-wing UAV’s maximum endurance calculation. The battery mass equation must first be rearranged to solve for power:

(68) $$P=\frac{m_{B}E_{D}}{t}$$

The calculations will be conducted based on the assumption of using Li-ion batteries with the highest energy density, which is about 265 Wh/kg [20]. The amount of available power in the battery depends on the battery mass, so a mass of 1 kg will be assumed as well, as this is also a common battery mass. The time is already known to be about an hour, which will be plugged in as 3600 s. Based on these values, the power used by the Coyote is calculated as follows:

(69) $$P=\frac{\left(1\;kg\right)\left(954,000\frac{Ws}{kg}\right)}{3600\;s}=265\;W$$

From this calculation alone, it is clear that the Coyote uses up much more power than is required for the smart-wing UAV system design. Even at the lowest altitude, which requires the most power, this UAV system design uses less power because of the fluidity in the design. To further prove the improvements in efficiency, the endurance for each airfoil will be calculated, and from this, the ranges will be determined as well. The results are provided in Table 12. Seconds were used as the unit of time in the calculations. However, they were then converted to minutes and rounded to the nearest minute for the clearest demonstration. Additionally, meters were used as the units of range and were converted into miles and rounded to the nearest mile.

The comparison between the disposable UAV system with shape-changing wings and the Coyote UAV system shows significant performance improvements in terms of flight duration, distance, and altitude capability. The power calculations for the variable wing area design demonstrate that the power required remains constant at each desired altitude, resulting in an endurance of approximately 119 min and a range of nearly 111 miles. These results indicate the efficiency of the design compared to that of the Coyote, offering different benefits. The variable wing area design is more suitable for lower altitude missions, while the airfoil-changing design is more suitable for higher altitudes. This provides two design options that outperform the Coyote, allowing for flexibility for designers and customers to choose according to their specific requirements.

## 4. Four-Dimensional Printing vs. Other Manufacturing Methods

Using biodegradable shape memory polymers for disposable UAV wings yields performance benefits; 4D printing, compared to traditional methods such as CNC machining, offers automated production, enhanced accuracy, and minimal waste. Multi-material printing enhances wing design, reducing errors and improving performance; 4D printing simplifies morphable wing production, optimizing cost, customization, and structural integrity. This innovation revolutionizes UAV wing manufacturing, boosting flexibility, efficiency, and performance.

## 5. Limitations of This Study

The study acknowledges valid concepts for enhancing the flight characteristics and manufacturing simplicity of disposable UAVs. However, challenges exist, including the preliminary calculations used; the weather conditions, which may alter the temperature–altitude relation, and the shape memory polymer limitations, which may impact accuracy and efficiency. The 3D printing size and material constraints, though restricting, show potential and advantages compared to other methods.

## 6. Conclusions

The study observed and analyzed the disposable UAV wing design process to enhance UAV flight characteristics and manufacturing efficiency. Introducing biodegradable shape memory polymers into wing design eliminated the need for actuators. Employing 4D printing as the manufacturing method simplified the process further by reducing or eliminating manual labor. Biodegradable SMPs improved UAV flight in several ways. Wing shapes were programmed to match altitude ranges and respond to the environment, preventing human error and enhancing efficiency. The disposable UAV system maintained cruising speeds across altitudes without increased power demand, leading to improved endurance and range for more efficient flight.

## Figures and Tables

**Table 1:** Statistical values of maximum lift coefficient and stall speed for different types of aircraft [16].

Aircraft Type	$C_{L_{max}}$	*V_s_* (Knot)
Hang glider/kite	2.5–3.5	10–15
Sailplane/glider	1.8–2.5	12–25
Microlight	1.8–2.4	20–30
Very light	1.6–2.2	30–45
GA light	1.6–2.2	40–61
Agricultural	1.5–2	45–61
Home-built	1.2–1.8	40–70
Business jet	1.6–2.6	70–120
Jet transport	2.2–3.2	95–130
Supersonic fighter	1.8–3.2	100–120

**Table 2:** Statistical values of wing aspect ratios for different types of aircraft [16].

Aircraft Type	Aspect Ratio
Hang glider	4–9
Glider (sailplane)	20–40
Home-built	4–7
General aviation	5–9
Jet trainer	4–8
Low-subsonic transport	6–9
High-subsonic transport	8–12
Supersonic fighter	2–4
Tactical missile	0.3–1
Hypersonic aircraft	1–3

**Table 3:** Statistical values of zero-lift drag coefficients for different types of aircraft [16].

Aircraft Type	$C_{D_{o}}$
Jet transport	0.015–0.02
Turboprop transport	0.018–0.024
Twin-engine piston prop	0.022–0.028
Small GA with fixed landing gear	0.02–0.03
Small GA with fixed landing gear	0.025–0.04
Agricultural	0.04–0.07
Sailplane/glider	0.012–0.015
Supersonic fighter	0.018–0.035
Home-built	0.025–0.04
Microlight	0.02–0.035

**Table 4:** Statistical values of maximum lift-to-drag ratios for different types of aircraft [16].

Aircraft Type	(*L/D*)_*max*_
Sailplane (glider)	20–35
Jet transport	12–20
GA	10–15
Subsonic military	8–11
Supersonic fighter	5–8
Helicopter	2–4
Home-built	6–14
Ultralight	8–15

**Table 5:** Statistical values of friction coefficients for different types of surfaces [16].

Surface	Friction Coefficient (*μ*)
Dry concrete/asphalt	0.03–0.05
Wet concrete/asphalt	0.05
Icy concrete/asphalt	0.02
Turf	0.04–0.07
Grass	0.05–0.1
Soft ground	0.1–0.3

**Table 6:** Air densities and design lift coefficients for various altitudes.

Altitude (ft)	Air Density (kg/m^3^)	Design Lift Coefficient
0	1.225	0.2903
1000	1.1895	0.2989
5000	1.0555	0.3369
10,000	0.9050	0.3929
15,000	0.7710	0.4612
20,000	0.6530	0.5445
25,000	0.5494	0.6472
30,000	0.4592	0.7743
35,000	0.3803	0.9350
40,000	0.3025	1.1754

**Table 7:** Air densities and altitudes associated with each design lift coefficient.

Design Lift Coefficient	Air Density (kg/m^3^)	Altitude (ft)
0.05	7.1114	N/A
0.15	2.3705	N/A
0.3	1.1852	1123.6877
0.45	0.7902	14,245.41
0.6	0.5926	22,837.93
0.75	0.4741	29,117.45
0.9	0.3951	34,009.186

**Table 8:** Altitude ranges for each NACA airfoil.

NACA Airfoil	Altitude Range (ft)
25015	0 to 1123.6877
35015	1123.6877 to 14,245.41
45015	14,245.41 to 22,837.93
55015	22,837.93 to 29,117.45
65015	29,117.45 to 34,009.186

**Table 9:** Design lift coefficients and their relevant parameters.

Design Lift Coefficient	Altitude (ft)	Air Density (kg/m^3^)	Temperature (°C)	Dynamic Pressure (Pa)	Force (N)
0.3	0	1.225	15	382.8125	179.3094
0.45	1123.6877	1.1852	12.8	370.375	173.4837
0.6	14,245.41	0.7902	−13.2	246.9375	115.6655
0.75	22,837.93	0.5926	−30.2	185.1875	86.7412
0.9	29,117.45	0.4741	−42.6	148.1563	69.3964

**Table 10:** Wing areas, wingspans, and other parameters associated with each cruising altitude.

Cruising Altitude (ft)	Air Density at Cruising Altitude (kg/m^3^)	Relative Density of Cruising Altitude	Maximum Altitude (ft)	Air Density at Maximum Altitude (kg/m^3^)	Relative Density at a Maximum Altitude	Wing Planform Area (m^2^)	Wingspan (m)
10,000	0.9050	0.7388	12,000	0.8493	0.6933	0.5463	2.3588
15,000	0.7710	0.6294	18,000	0.6985	0.5702	0.6413	2.7690
20,000	0.6530	0.5331	24,000	0.5692	0.4647	0.7572	3.2694
25,000	0.5494	0.4485	30,000	0.4592	0.3749	0.9000	3.8860
30,000	0.4592	0.3749	36,000	0.3661	0.2989	1.0768	4.6494
35,000	0.3803	0.3104	42,000	0.2750	0.2245	1.3001	5.6136

**Table 11:** Lift, drag, and power results for each airfoil.

Airfoil	Lift Coefficient	Drag Coefficient	Drag Force (N)	Power (W)
25015	0.2565	0.0330	5.7250	143.125
35015	0.3847	0.0394	4.5572	113.93
45015	0.5130	0.0459	3.9814	99.535
55015	0.6412	0.0524	3.6364	90.91
65015	0.7695	0.0589	3.4063	85.1575

**Table 12:** Endurance and range for each airfoil.

Airfoil	Endurance (min)	Range (mi)
25015	111	103
35015	140	130
45015	160	149
55015	175	163
65015	187	174

## Data Availability

Not applicable.
